# Purpureocillium lilacinum Keratitis and Endophthalmitis in an Aphakic Eye With Secondary Glaucoma: A Case Report

**DOI:** 10.7759/cureus.99499

**Published:** 2025-12-17

**Authors:** Nikolina Tsirogianni, Anne Maria Walch, Maged Alnawaiseh, Olaf Kaup, Robin Stuempeley

**Affiliations:** 1 Ophthalmology, Klinikum Bielefeld (Universitätsklinikum OWL), Bielefeld, DEU; 2 Microbiology, Klinikum Bielefeld (Universitätsklinikum OWL), Bielefeld, DEU

**Keywords:** antifungal therapy, endophthalmitis, fungal keratitis, paecilomyces lilacinus, purpureocillium lilacinum

## Abstract

*Purpureocillium lilacinum*(formerly *Paecilomyces lilacinus*) is a rare filamentous fungus that can cause keratitis in eyes with predisposing factors such as chronic ocular surface disease, previous surgery, trauma, or long-term use of topical steroids. This infection often exhibits resistance to standard antifungal agents and can result in severe visual loss. We report a case of keratitis combined with endophthalmitis in a male patient under 60 years of age with a history of childhood ocular trauma leading to aphakia and secondary glaucoma of the right eye. The patient had undergone several previous interventions, including cyclocryotherapy for glaucoma control. He presented with progressive corneal whitening, pain, and marked vision loss. On admission, the right eye showed a dense central corneal ulcer with full-thickness stromal infiltration, hypopyon, and vitreous opacities. The cornea was opaque, preventing a clear view of the anterior chamber or fundus. The first corneal swab showed no microbial growth. Subsequent cultures of conjunctival and corneal swabs yielded *P. lilacinum*. Intensive topical and systemic therapy with voriconazole, natamycin, vancomycin, and ceftazidime was initiated. Enucleation was recommended due to pain, poor prognosis, and early signs of phthisis on ocular B-scan, but the patient initially refused. The eye remained anatomically intact but functionally blind. At a later emergency follow-up, enucleation was eventually accepted. *P. lilacinum* keratitis is challenging to diagnose and manage because of its resistance to amphotericin B and natamycin. Voriconazole demonstrates the greatest efficacy, but visual prognosis is often poor once deep stromal invasion occurs. Repeated cultures may be necessary for diagnosis, as initial samples may show no growth. Early antifungal therapy and clear communication with the patient are essential. This case illustrates the therapeutic challenges of *P. lilacinum* keratitis and the rapid progression of the disease despite treatment. Even with appropriate antifungal therapy, structural damage and vision loss may be irreversible. Early microbiological diagnosis and honest discussion with the patient regarding the poor prognosis are critical.

## Introduction

Fungal keratitis is a corneal infection caused by yeasts (e.g., the genus *Candida*) or filamentous fungi (e.g., the genera *Fusarium *and *Aspergillus*). It can elicit a severe inflammatory response and is often associated with poor visual prognosis [1]. *Purpureocillium lilacinum *is a ubiquitous environmental fungus found in soil, decaying organic matter, and water [2]. Formerly classified as *Paecilomyces lilacinus*, it was redefined based on molecular phylogeny in 2011 [3]. Human infection is uncommon and usually occurs in immunocompromised hosts or in eyes with predisposing ocular surface disease. Keratitis is rare but may follow trauma, surgery, or prolonged corticosteroid use [3-5].

Treatment is often challenging due to the organism’s resistance to amphotericin B and natamycin [6,7], with voriconazole being a more effective alternative [6,8]. Nevertheless, even with appropriate therapy, functional recovery is frequently limited [4,9]. This case highlights diagnostic delays, resistance-related treatment challenges, and poor outcomes that can occur despite early initiation of voriconazole, underscoring the importance of maintaining a high index of suspicion for *P. lilacinum *keratitis in surgically altered eyes.

## Case presentation

A male patient under 60 years of age with a history of childhood trauma to the right eye presented in 2015 with aphakia, iris coloboma, and secondary glaucoma. Multiple prior interventions had been performed, including scleral cerclage and cyclocryotherapy. The left eye remained normal. At that time, visual acuity in the right eye was 20/200, and ocular hypertension (45 mmHg) was successfully managed with topical medications. The patient was subsequently lost to follow-up for several years.

**Figure 1:**
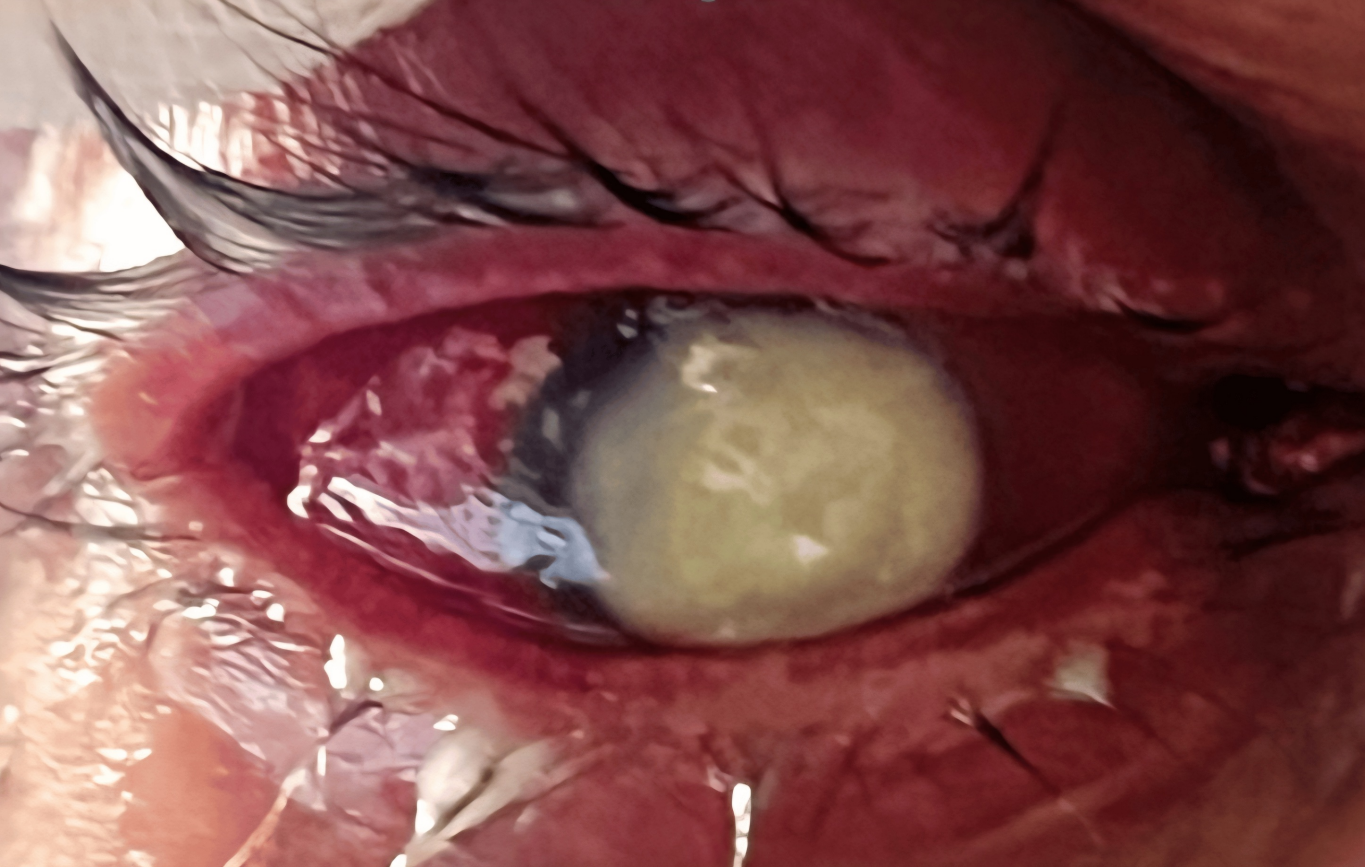
Right eye at initial presentation (September 2025)

In 2024, he re-presented with progressive elevation of intraocular pressure, requiring cyclocryotherapy, which resulted in temporary pressure control (15 mmHg postoperatively). The patient was again lost to follow-up. In September 2025, he presented with severe ocular pain, photophobia, and complete vision loss (visual acuity: light perception). Examination revealed a dense central corneal ulcer with total stromal infiltration, hypopyon, and vitreous opacity (Figure 1).

The patient’s clinical course and key management milestones are summarized in Table 1. 

**Table 1:** Summary of clinical timeline IOP, intraocular pressure

Date	Event
Childhood	Ocular trauma → aphakia, secondary glaucoma
2015	Re-presentation; IOP 45 mmHg – controlled with topical therapy
2015-2024	Lost to follow-up
2024	Elevated IOP → cyclocryotherapy → temporary control
2024-2025	Lost to follow-up again
September 2025	Severe pain, light perception vision, dense corneal ulcer, hypopyon
Early October 2025	Initial cultures negative; empirical therapy initiated
Late October 2025	Cultures positive for *Purpureocillium lilacinum*; targeted therapy initiated
Late October 2025	Early phthisis on B-scan; enucleation recommended
November 2025	Emergency enucleation performed

Initial cultures were negative. Empirical topical antibiotics were initiated, including moxifloxacin, gentamicin, neomycin sulfate, gramicidin, and polymyxin B sulfate, along with ongoing management of glaucoma and systemic hypertension.

**Figure 2:**
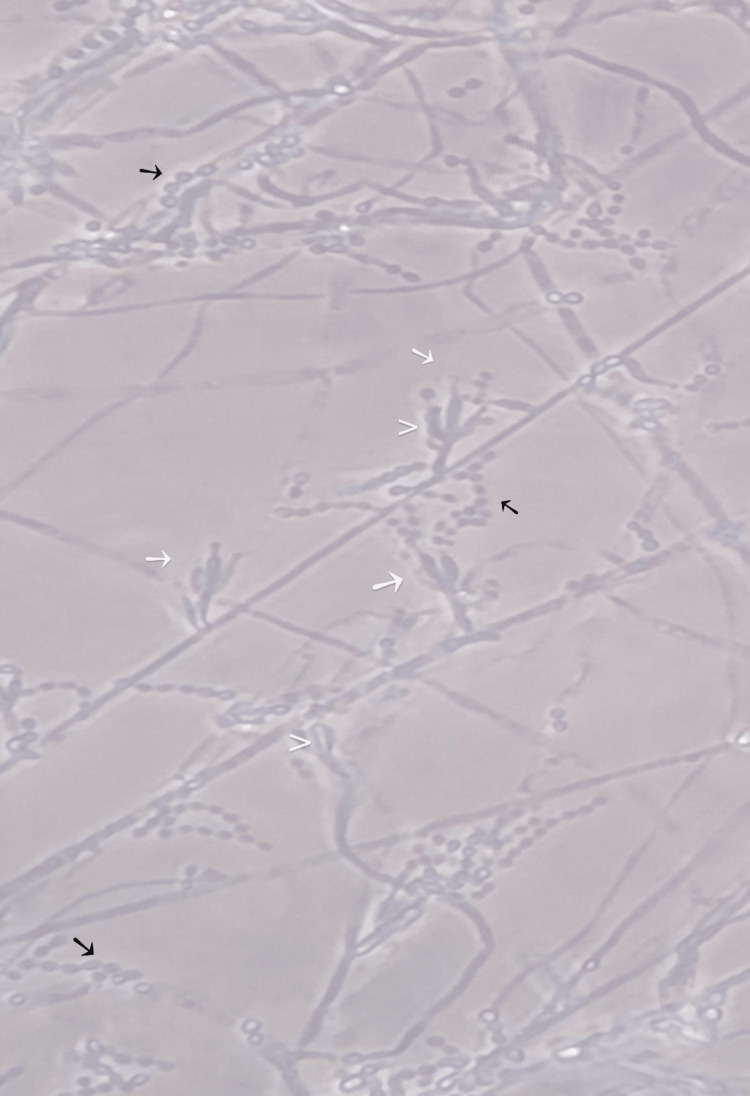
Native culture microscopy using lactophenol cotton blue stain (×400, phase contrast) showing finger-shaped, upright conidiophores with rough walls (white arrow), densely packed phialides (arrowhead), hyaline mycelium, and numerous elliptical conidia (black arrow) arranged in chains

**Figure 3:**
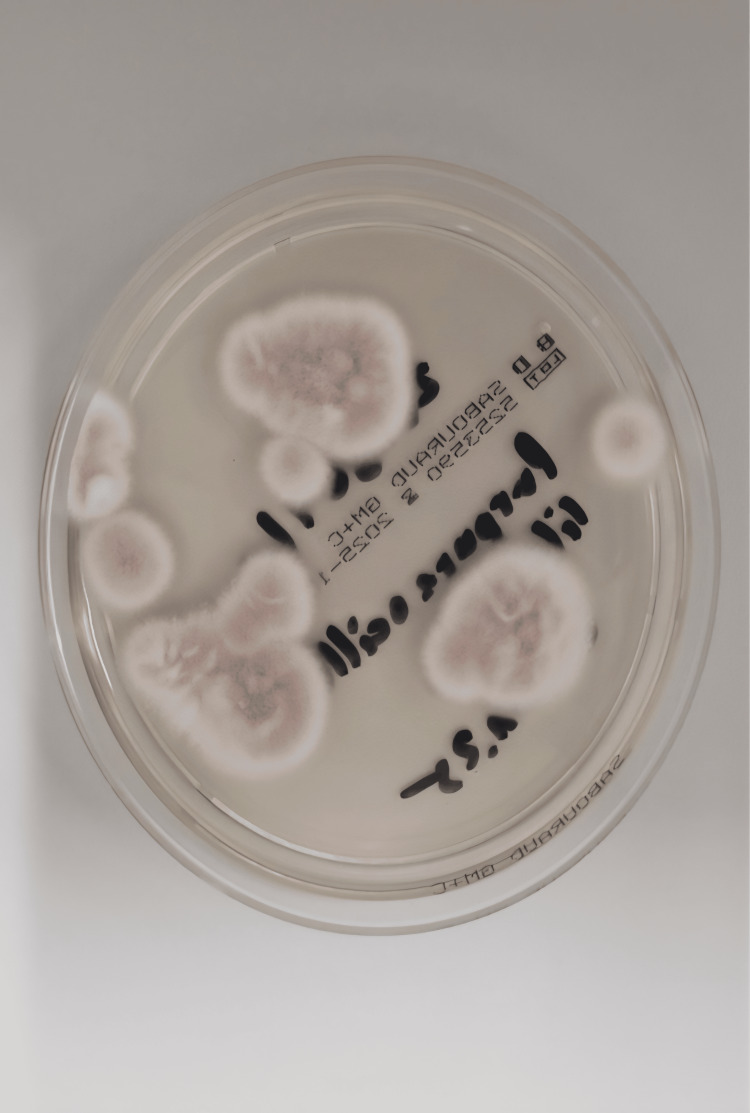
Colony surface of Purpureocillium lilacinum on Sabouraud glucose agar with gentamicin and chloramphenicol, demonstrating a distinctly developed pearl-pink to wine-red pigment with a lighter, well-defined growth margin

On readmission in October 2025, the infection had progressed. Several corneal swabs were obtained, and all initial microbiological results remained negative. However, the most recent swab subsequently yielded growth after a few days, confirming *P. lilacinum* (Figure 2, Figure 3).

Intensive therapy was initiated with topical voriconazole 2%, natamycin, vancomycin 5%, and ceftazidime 2%, combined with systemic oral voriconazole (loading dose of 400 mg for two days, followed by 200 mg daily). Despite treatment, the cornea remained opaque with no view of the anterior chamber, and ocular B-scan revealed early signs of phthisis bulbi. Enucleation was recommended, but the patient initially refused and opted to continue conservative therapy.

**Figure 4:**
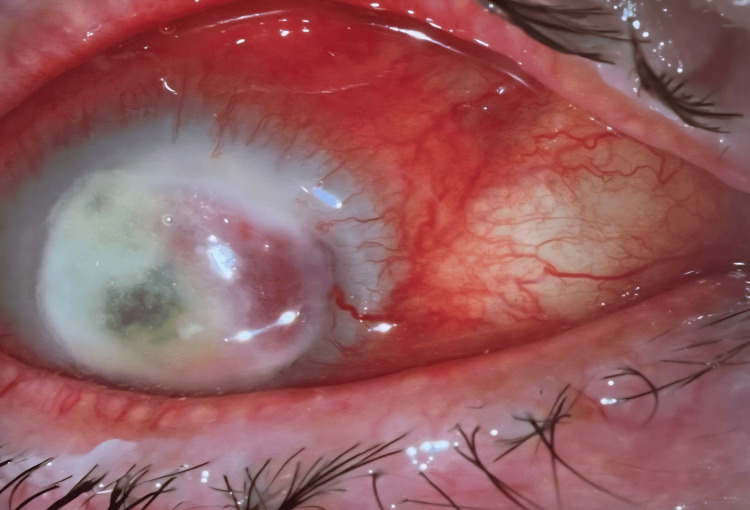
Right eye at emergency presentation, three weeks after discharge following initial refusal of enucleation (after completion of voriconazole therapy)

Approximately three weeks later, the patient re-presented to the emergency department (Figure 4) with increasing pain and chose to proceed with enucleation, which was subsequently performed.

## Discussion

*P. lilacinum* keratitis is an uncommon but increasingly recognized cause of fungal keratitis, particularly in eyes with preexisting structural abnormalities, prior surgery, trauma, or prolonged topical corticosteroid use [2-4]. Diagnosis may be delayed due to its indolent clinical course and frequently negative initial cultures [5]. The organism’s low virulence and slow growth often contribute to delayed detection, as early corneal cultures may fail to yield growth [5]. This was evident in our case, where multiple early cultures were negative, postponing targeted antifungal therapy.

A major challenge in managing *P. lilacinum *infections is its intrinsic resistance to many commonly used antifungal agents, including amphotericin B and, in some cases, natamycin [6,7]. Voriconazole is currently considered the treatment of choice [6,8]. Nevertheless, even with early initiation of voriconazole, treatment failure and poor visual outcomes remain common, especially when deep stromal invasion or intraocular extension occurs, as observed in our patient [4,8].

Natamycin was continued briefly after identification of *P. lilacinum *because broad-spectrum antifungal coverage remained clinically necessary in the setting of rapidly progressive keratitis. Although *P. lilacinum *demonstrates intrinsic resistance to natamycin, abrupt discontinuation of a frontline topical antifungal in a severely compromised eye could have permitted further deterioration before voriconazole reached full therapeutic levels. Additionally, natamycin provided prophylactic coverage against other filamentous fungi, which remained a consideration given the initially negative cultures. Once clinical nonresponse was confirmed, natamycin was discontinued.

B-scan ultrasonography played a key role in clinical decision-making. Early phthisical changes, including posterior segment collapse and reduced globe integrity, indicated a poor structural prognosis despite intensive antifungal therapy. These findings, together with persistent pain and lack of visual potential, supported the recommendation for enucleation. The patient’s initial refusal likely contributed to further deterioration until emergency enucleation became necessary.

Our patient presented with several well-established risk factors, including aphakia, a history of multiple intraocular surgeries, chronic ocular surface instability, and secondary glaucoma [4,5]. These factors likely facilitated both the initial infection and rapid disease progression despite aggressive antifungal therapy. Aphakia may have allowed easier posterior segment involvement, predisposing the patient to endophthalmitis and early phthisis bulbi.

The progression to functional blindness, despite combined intensive topical and systemic antifungal therapy, highlights the aggressive nature of advanced *P. lilacinum* infections [4,9]. In recalcitrant cases, surgical intervention, including therapeutic keratoplasty or enucleation, may be required to control infection and relieve pain [4,8].

Emerging antifungal agents may offer future therapeutic options for *P. lilacinum *keratitis. Novel triterpenoids such as ibrexafungerp and orotomide-class agents such as olofim have shown promising activity against resistant molds in preclinical studies, although clinical data in ophthalmic infections remain limited. These developments emphasize the need for antifungals with improved corneal penetration and reliable activity against organisms with intrinsic resistance to conventional therapy [10].

This case also underscores the importance of early and transparent communication with patients regarding prognosis and potential surgical outcomes. Younger patients, in particular, may be reluctant to consent to enucleation, even when visual potential is lost. Clear counseling is therefore essential to avoid prolonged suffering and repeated hospitalizations.

A limitation of this report is the absence of antifungal susceptibility testing and post-enucleation histopathology, which could have provided additional microbiological and structural confirmation.

In summary, *P. lilacinum* keratitis should be suspected in cases of nonhealing keratitis with repeatedly negative cultures, particularly in eyes with prior surgery or chronic ocular surface disease [2,5]. The epidemiology of fungal keratitis varies worldwide, with higher incidence and more severe presentations reported in developing regions [9,11]. Early microbiological diagnosis, prompt initiation of voriconazole-based therapy, and timely surgical decision-making are critical for limiting ocular morbidity [6,8,11].

## Conclusions

*P. lilacinum* keratitis is an uncommon but potentially devastating fungal infection that may be resistant to standard antifungal therapy. Rapid microbiological diagnosis and early initiation of targeted antifungal treatment are essential to preserve ocular integrity. Clinicians should maintain a high index of suspicion for this organism in cases of recalcitrant keratitis, particularly in surgically altered eyes.
